# Associations Between *hOGG1* Ser326Cys Polymorphism and Increased Body Mass Index and Fasting Glucose Level in the Japanese General Population

**DOI:** 10.2188/jea.JE20140002

**Published:** 2014-09-05

**Authors:** Megumi Hara, Kazuyo Nakamura, Hinako Nanri, Yuichiro Nishida, Asahi Hishida, Sayo Kawai, Nobuyuki Hamajima, Yoshikuni Kita, Sadao Suzuki, Eva Mariane Mantjoro, Keizo Ohnaka, Hirokazu Uemura, Daisuke Matsui, Isao Oze, Haruo Mikami, Michiaki Kubo, Hideo Tanaka

**Affiliations:** 1 Department of Preventive Medicine, Faculty of Medicine, Saga University, Saga, Japan; 1 佐賀大学医学部社会医学講座予防医学; 2 St. Mary’s College, Faculty of Nursing, Kurume, Japan; 2 聖マリア学院大学看護学部; 3 Department of Public Health, Showa University, Tokyo, Japan; 3 昭和大学医学部衛生学公衆衛生学講座公衆衛生学; 4 Department of Preventive Medicine, Nagoya University Graduate School of Medicine, Nagoya, Japan; 4 名古屋大学大学院医学系研究科予防医学; 5 Department of Healthcare Administration, Nagoya University Graduate School of Medicine, Nagoya, Japan; 5 名古屋大学大学院医学系研究科社会生命科学講座医療行政学; 6 Department of Health Science, Shiga University of Medical Science, Otsu, Japan; 6 滋賀医科大学公衆衛生学; 7 Department of Public Health, Nagoya City University Graduate School of Medical Sciences, Nagoya, Japan; 7 名古屋市立大学大学院医学研究科公衆衛生学; 8 Department of International Island and Community Medicine, Kagoshima University Graduate School of Medical and Dental Science, Kagoshima, Japan; 8 鹿児島大学大学院医歯学総合研究科国際島嶼医療学; 9 Department of Geriatric Medicine, Kyushu University Graduate School of Medical Sciences, Fukuoka, Japan; 9 九州大学大学院医学研究院予防医学; 10 Department of Preventive Medicine, Institute of Health Biosciences, University of Tokushima Graduate School, Tokushima, Japan; 10 徳島大学大学院ヘルスバイオサイエンス研究部予防医学; 11 Department of Epidemiology for Community Health and Medicine, Kyoto Prefectural University of Medicine, Kyoto, Japan; 11 京都府立医科大学大学院医学研究科地域保健医療疫学; 12 Division of Epidemiology and Prevention, Aichi Cancer Center Research Institute, Nagoya, Japan; 12 愛知県がんセンター研究所疫学・予防部; 13 Division of Cancer Registry, Prevention and Epidemiology, Chiba Cancer Center, Chiba, Japan; 13 千葉県がんセンター研究局がん予防センター; 14 Laboratory for Genotyping Development, Center for Genomic Medicine, RIKEN, Yokohama, Japan; 14 理化学研究所ゲノム医科学研究センター

**Keywords:** human 8-oxoguanine glycosylase 1 (*hOGG1*), obesity, body mass index (BMI), fasting blood glucose (FBG), polymorphism, study area

## Abstract

**Background:**

Evidence suggests that Ser326Cys, a genetic polymorphism of human 8-oxoguanine glycosylase 1 (*hOGG1*), is associated with insulin resistance and type 2 diabetes; however, the underlying mechanism is unclear. Recently, an animal study showed a significant association between the *hOGG1* genotype and obesity, although evidence for such an association in humans is limited. The purpose of this study was to examine the association between the *hOGG1* genotype and body mass index (BMI) and fasting blood glucose (FBG) levels.

**Methods:**

Cross-sectional analysis was conducted using the baseline survey data from a Japan Multi-Institutional Collaborative Cohort Study, which included 1793 participants aged 40–69 years. The *hOGG1* polymorphism was detected using a multiplex polymerase chain reaction-based invader assay. Multiple linear regression, analysis of covariance, and logistic regression were used to control for confounding variables.

**Results:**

The Cys allele was significantly associated with increased BMI, FBG level, and total cholesterol (TC) level, even after adjustment for gender, age, energy intake, alcohol, smoking, physical activity, and family history of diabetes. An association with BMI was still observed after further adjustment for FBG and TC, but not for the study area (Amami or the mainland). The Cys/Cys genotype was significantly more prevalent in the participants with higher BMI (>27.5 kg/m^2^). However, the impact of genotype decreased and significance disappeared after adjusting for the study area.

**Conclusions:**

The present results suggest that the study area being inside Japan confounds the association between *hOGG1* genotype and obesity.

## Abstract (ja)

**背景：:**

DNA修復酵素であるヒト8―オキソグアニンDNAグリコシラーゼ *(hOGG1)*のSer326Cys遺伝子多型はインスリン抵抗性や２型糖尿病との関連が示唆されているが、その機序については明らかでない。近年、動物実験において*OGG1*の遺伝子多型と肥満の有意な関連が報告されたが、人での報告はほとんどない。本研究の目的は、*hOGG1*の遺伝子多型とBMIおよび空腹時血糖との関連を調べることである。

**方法：:**

日本多施設共同コーホート研究（ジェイミック　スタディ）に参加した40～69歳の男女1,793人の資料及び試料を用いて横断研究を実施した。*hOGG1*の遺伝子多型は、Multiplex PCR-based Invader assay法により決定した。重回帰分析、共分散分析、ロジスティック回帰分析を用いて交絡因子を調整し検討を行った。

**結果：:**

性、年齢、摂取エネルギー、飲酒、喫煙、身体活動量、糖尿病の家族歴を補正しても、Cysアレル保有者ではBMI、空腹時血糖、総コレステロールが有意に高い値を示した。BMIとCysアレルの有意な関連は、さらに空腹時血糖と総コレステロールの値を調整しても認められたが、調査地域（奄美地域か、それ以外の地域か）で調整すると有意な関連は検出されなくなった。Cys/Cys型では高いBMI（>27.5 kg/m^2^)との有意な関連がみられたが、調査地域を調整すると関連は弱まり、有意差も検出されなくなった。

**結論：:**

*hOGG1*の遺伝子多型と肥満の関連に対し、日本人に限った研究においても調査地域による交絡が存在することが示された。

## INTRODUCTION

Reactive oxygen species (ROS) are known to play an essential role in the pathogenesis of diabetes.^1^ Several studies have reported that oxidative stress associated with insulin resistance, β cell dysfunction, impaired glucose tolerance, and mitochondrial dysfunction can ultimately lead to the diabetes disease state.^2^^–^^4^ ROS also cause strand breaks and base modifications in DNA, including the oxidation of guanine residues to 8-hydroxy-2′-deoxyguanine (8-OHdG). These ROS-induced mutations alter the function of various genes and influence the pathogenesis of several diseases, such as cancer, cardiovascular disease, neurodegenerative diseases, and diabetes.^1^ Base-excision repair (BER) plays an important role in preventing such disease, and human 8-oxoguanine glycosylase 1 (*hOGG1*) is one of the key glycosylases involved in the BER system.^5^ The Ser326Cys polymorphism of the highly polymorphic OGG1 gene has been studied the most because this polymorphism is associated with functional differences in enzyme activity^6^ and loss of function.^7^ However, most epidemiological studies of this polymorphism have focused on cancer susceptibility.^8^^–^^12^

In the past decade, several studies have reported that the Ser326Cys *hOGG1* polymorphism is associated with insulin resistance^13^ and type 2 diabetes^14^^–^^17^; however, the underlying mechanism has not been elucidated. Obesity-associated insulin resistance is a major risk factor for type 2 diabetes,^18^ and fat accumulation has been reported to be associated with systemic oxidative stress^19^^,^^20^; therefore, it may be possible to assess the risk of diabetes based on the association between the Ser326Cys *hOGG1* polymorphism and body mass index (BMI). Recently, an animal study found that *hOGG1* deficiency alters cellular substrate metabolism, which favors a sparing phenotype and increased susceptibility to obesity^21^; however, evidence for such association between *hOGG1* polymorphism and BMI in humans is limited.^14^

The purpose of this study was to determine whether the *hOGG1* Cys allele is associated with BMI and fasting glucose level. We also studied whether this association is modified by the effects of other factors.

## METHODS

### Study participants

The purpose of the Japan Multi-Institutional Collaborative Cohort (J-MICC) Study is to confirm and detect gene-environment interactions for lifestyle-related diseases using a large genome cohort, as previously described.^22^ Briefly, the J-MICC Study, which was initiated in 2005, included volunteers aged 35–69 years from 10 areas of Japan: Chiba, Shizuoka, Okazaki, Aichi, Takashima and Kyoto, which are located in Honshu Island; Tokushima, which is located in Shikoku Island; Fukuoka and Saga, which are located in Kyushu Island; and Amami, which is located 380 km southwest of Kyushu Island. Throughout this paper, we refer to Honshu Island, Shikoku Island, and Kyushu Island as “the mainland”. In this cross-sectional study, data from 4512 participants throughout these areas were collected during the period of 2005–2008.^23^ Written informed consent was obtained from all participants. The study protocol was approved by the Nagoya University School of Medicine ethics committees and other participating institutions.

### Questionnaire and measurements

A self-administered questionnaire was used to collect data on alcohol consumption, smoking, dietary habits, physical activity, current medication, disease history, and first-degree family history of diabetes. Details of the dietary assessment and estimation of physical activity were reported elsewhere.^24^^–^^27^

Height and weight were measured to the nearest 0.1 cm and 0.1 kg, respectively. Body mass index (BMI) was calculated as the weight in kilograms divided by the square of the height in meters (kg/m^2^). We defined a BMI of >27.5 kg/m^2^ as obese; increased mortality has been reported above this point among East Asians.^28^ The HbA1c (%) and fasting blood glucose (FBG), triglyceride, total cholesterol, and HDL cholesterol levels were measured in laboratories in each study area, and the results of these measurements were collected. The HbA1c (%) value was converted from the Japan Diabetes Society (JDS) to the National Glycohemoglobin Standardization Program (NGSP) by using the following equation published by the JDS: NGSP (%) = 1.02 × JDS (%) + 0.25%.^29^

### Genotyping

Genotyping was performed as described previously.^23^ Single nucleotide polymorphisms, including the *hOGG1* Ser326Cys (rs1052133), were genotyped using a multiplex polymerase chain reaction-based Invader assay (Third Wave Technologies, Madison, WI, USA)^30^ at the Laboratory for Genotyping Development, Center for Genomic Medicine, RIKEN.

### Statistical analysis

In the analysis, we excluded 2719 participants based on any of the following conditions: missing data of *hOGG1* polymorphism (*n* = 11); missing FBG (*n* = 2627) data; taking type 2 diabetes medication (*n* = 89); or a dietary energy intake greater than 4000 kcal/day (*n* = 2). Consequently, data for 976 men and 817 women aged 35–69 years were retained for analysis. Among these participants, data on alcohol consumption (17 men and 23 women) or physical activity (5 men and 5 women) were missing for some participants.

All analyses were performed with the SAS statistical software package (Ver. 9.3 for Windows; SAS Institute, Cary, NC, USA). A *P* value of less than 0.05 was considered statistically significant.

To compare the characteristics of participants according to the *hOGG1* genotype, we used the Kruskal-Wallis test for continuous variables and χ^2^ tests for categorical variables. Adjusted means and their 95% confidence intervals (CIs) of BMI, FBG, and total cholesterol according to *hOGG1* genotype were evaluated by least-squares general linear regression, and linear trends were assessed by the statistical significance of the regression coefficient of an ordinal variable for the factor under the following considerations: gender; age (continuous); energy intake (continuous); physical activity level (continuous); alcohol consumption status (never, former, or current drinker consuming 0.1–22.9, 23.0–45.9, or ≥46.0 g ethanol/day); smoking status (never, former, or current smoker of 1–19, 20–39, or ≥40 cigarettes/day); first-degree family history of diabetes (positive, negative, or unknown); study area (Amami or the mainland); BMI (continuous, for the evaluation of FBG and total cholesterol); FBG (continuous, for BMI and total cholesterol); and total cholesterol (continuous, for BMI and FBG). Odds ratios (ORs) and 95% CIs of *hOGG1* genotype for excessive BMI (>27.5 kg/m^2^) were estimated using logistic regression models adjusted for potential confounders (age, BMI, energy intake, alcohol consumption, smoking, physical activity, family history of diabetes, and study area).

## RESULTS

The characteristics of study participants according to the *hOGG1* Ser326Cys genotype are shown in Table 1. The genotype distributions of the *hOGG1* Ser326Cys gene among all participants followed the Hardy-Weinberg equilibrium (χ^2^ = 0.511, *P* = 0.475). Genotype frequency was significantly different in the Amami area (*P* < 0.001). The Cys allele carriers had significantly higher mean BMI (*P* = 0.021) and FBG (*P* < 0.001) levels, and a higher proportion were current alcohol drinkers (*P* < 0.001). TC level tended to be higher in Cys allele carriers, although this difference was not statistically significant (*P* = 0.077).

**Table 1.** Characteristics according to the *hOGG1* Ser326Cys genotype among 1793 subjects

	Ser/Ser		Ser/Cys		Cys/Cys		*P*
*n* (%) *n* = 1793	365	(20.4)	866	(48.3)	562	(31.3)	
Gender, women (%)	180	(49.3)	393	(45.4)	244	(43.4)	0.209
Age (y) (SD)	55.0	(8.6)	54.5	(8.9)	55.1	(8.7)	0.354
Study area, Amami area (%)	60	(16.4)	185	(21.4)	204	(36.3)	<0.001
Total energy intake (kcal/d) (SD)	1729.7	(348.4)	1752.9	(371.6)	1730.0	(376.3)	0.406
BMI (kg/m^2^) (SD)	23.2	(3.0)	23.2	(3.3)	23.7	(3.5)	0.021
Physical activity level (METs·h) (SD)	13.6	(12.1)	14.7	(13.9)	15.5	(14.9)	0.775
Current alcohol drinkers, *n* (%)	192	(53.6)	485	(57.2)	333	(60.9)	0.001
Current smoking, *n* (%)	63	(17.3)	155	(17.9)	98	(17.4)	0.981
Family history of diabetes, *n* (%)	58	(15.9)	142	(16.4)	85	(15.1)	0.451
HbA1c (NGSP) (%)	5.61	(0.55)	5.54	(0.44)	5.58	(0.46)	0.199
FBG (mg/dL) (SD)	96.9	(13.3)	96.6	(14.6)	99.4	(16.9)	<0.001
TG (mg/dL) (SD)	110.8	(69.1)	114.0	(80.9)	116.7	(82.4)	0.368
TC (mg/dL) (SD)	207.1	(33.0)	210.9	(34.0)	212.3	(34.2)	0.077
HDL-C (mg/dL) (SD)	64.7	(16.5)	64.1	(16.4)	63.3	(16.5)	0.278

BMI, body mass index; FBG, fasting blood glucose; TG, triglyceride; TC, total cholesterol; HDL-C, high-density lipoprotein cholesterol.

*P* values for Chi-square test or Kruskal-Wallis test.

After adjusting for possible confounding factors, such as gender, age, energy intake, physical activity level, ethanol intake, smoking, and family history of diabetes, the Cys allele was found to be significantly associated with higher BMI, FBG, and TC levels in a dose-dependent manner (all *P* < 0.05, Table 2, Model 2). The association with BMI was still significant after further adjustment for FBG and TC (*P* = 0.02, Model 3). However, the significance disappeared after adjusting for study area (*P* = 0.23, Model 4).

**Table 2.** Adjusted means of BMI, FBG, and TC according to *hOGG1* Ser326Cys genotype

	Ser/Ser		Ser/Cys		Cys/Cys		*P* for trend
		
	Mean	95% CI	Mean	95% CI	Mean	95% CI	
Model 1^a^							
BMI (kg/m^2^)	23.2	(22.8–23.5)	23.2	(23.0–23.4)	23.7	(23.4–24.0)	0.013
FBG (mg/dL)	97.1	(95.6–98.6)	96.6	(95.6–97.6)	99.2	(98.0–100.4)	0.013
TC (mg/dL)	206.6	(203.2–210.0)	210.9	(208.6–213.1)	212	(209.5–215.0)	0.025
Model 2^b^							
BMI (kg/m^2^)	23.2	(22.9–23.5)	23.2	(23.0–23.4)	23.7	(23.4–24.0)	0.018
FBG (mg/dL)	97.3	(95.8–98.8)	96.3	(95.4–97.2)	99.1	(97.9–100.2)	0.025
TC (mg/dL)	207.0	(203.5–210.4)	210.8	(208.5–213.0)	212	(209.1–214.6)	0.040
Model 3^c^							
BMI (kg/m^2^)	23.2	(22.8–23.5)	23.3	(23.1–23.5)	23.6	(23.4–23.9)	0.020
FBG (mg/dL)	97.7	(96.4–99.1)	96.6	(95.7–97.5)	98.4	(97.3–99.5)	0.260
TC (mg/dL)	207.1	(203.6–210.5)	211.0	(208.7–213.2)	212	(208.7–214.3)	0.071
Model 4^d^							
BMI (kg/m^2^)	23.3	(22.9–23.6)	23.3	(23.1–23.5)	23.5	(23.2–23.8)	0.230
FBG (mg/dL)	98.1	(96.7–99.4)	96.7	(95.8–97.6)	98.1	(97.0–99.2)	0.769
TC (mg/dL)	207.1	(203.7–210.6)	211.0	(208.7–213.2)	211	(208.6–214.3)	0.078

BMI, body mass index; CI, confidence interval; FBG, fasting blood glucose; TC, total cholesterol.

^a^Adjusted for gender and age (continuous).

^b^Adjusted for Model 1 and further adjusted for energy intake (continuous), physical activity level (continuous), ethanol intake (never, former, or current drinker consuming 0.1–22.9, 23.0–45.9, or ≥46 g ethanol/day), smoking (never, former, or current smoker of 1–19, 20–39, or ≥40 cigarettes/day), and family history of diabetes (positive, negative, or unknown).

^c^Adjusted for all variables in Model 2 and further adjusted for BMI (for FBG and TC), FBG (for BMI and TC), and TC (for BMI and FBG).

^d^Adjusted for all variables in Model 3 and further adjusted for the study area (Amami or the mainland).

Data for the evaluation of the association between obesity (BMI > 27.5 kg/m^2^) and *hOGG1* genotype using logistic regression analysis are shown in Table 3. The prevalence of obesity in the Cys/Cys genotype was significantly greater after adjusting for gender, age, energy intake, physical activity level, ethanol intake, smoking, family history of diabetes, FBG, and TC (Model 3). Although Cys allele carriers tended to have a higher proportion of obesity, the significance of this association disappeared after adjusting for the study area (Model 4). The OR of the Amami area for obesity was 2.44 (95% CI 1.67–3.56), which was greater than that of the *hOGG1* genotype.

**Table 3.** Odds ratios and 95% CIs for obesity (BMI > 27.5 kg/m^2^) according to *hOGG1* Ser326Cys genotype among 1793 subjects

BMI (kg/m^2^)	>27.5	≤27.5	Model 1^a^		Model 2^b^		Model 3^c^		Model 4^d^	
			
	*n*	*n*	OR	95% CI	OR	95% CI	OR	95% CI	OR	95% CI
Ser/Ser	24	341	1.00	reference	1.00	reference	1.00	reference	1.00	reference
Ser/Cys	84	782	1.50	(0.94–2.41)	1.44	(0.90–2.32)	1.55	(0.94–2.57)	1.45	(0.87–2.41)
Cys/Cys	62	500	1.77	(1.08–2.90)	1.74	(1.06–2.86)	1.74	(1.03–2.94)	1.46	(0.86–2.48)
			*P*_trend_ = 0.026		*P*_trend_ = 0.029		*P*_trend_ = 0.049		*P*_trend_ = 0.225	

BMI, body mass index; CI, confidence interval; OR, odds ratio.

^a^Adjusted for gender and age (continuous).

^b^Adjusted for Model 1 and further adjusted for energy intake (continuous), physical activity level (continuous), ethanol intake (never, former, or current drinker consuming 0.1–22.9, 23.0–45.9, or ≥46 g ethanol/d), smoking (never, former, or current smoker of 1–19, 20–39, or ≥40 cigarettes/d), and family history of diabetes (positive, negative, or unknown).

^c^Adjusted for all variables in Model 2 and further adjusted for FBG and TC.

^d^Adjusted for all variables in Model 3 and further adjusted for the study area (Amami or the mainland).

## DISCUSSION

In this cross-sectional study, we observed significant associations between the *hOGG1* Cys/Cys genotype and higher BMI and incidence of obesity, after adjustment for possible confounding factors other than the study area. After adjusting for study area, however, this significance disappeared, suggesting that study area is a confounding factor. Despite this lack of association, Cys allele carriers tended to have a higher proportion of obesity than Ser/Ser.

Several epidemiological studies have examined the associations between the Ser326Cys *hOGG1* polymorphism and insulin resistance^13^ and type 2 diabetes.^14^^–^^17^ Wang et al reported that the Cys/Cys variant significantly decreases insulin sensitivity, even after adjustment for possible confounders, including BMI, among the Taiwanese.^13^ Three studies detected significant association between Ser326Cys *hOGG1* polymorphism and diabetes.^14^^,^^16^^,^^17^ Specifically, Daimon et al reported that decreased insulin secretion is associated with being a Cys allele carrier, measured by homeostatic model assessment beta cell function (HOMA-β) in a Japanese population,^14^ while Gönül et al reported a significant association between the Cys allele and insulin resistance in a Turkish population, measured by HOMA-R.^17^ On the other hand, one case-control study conducted in a Polish population failed to detect an association between this polymorphism and diabetes^15^ due to limited sample size. Regarding obesity, an animal study showed significant association between the *hOGG1* genotype and obesity,^21^ and one epidemiological study reported a positive association between BMI and this polymorphism.^14^ Our study showed no evidence of an association between this polymorphism and increased BMI and FBG.

In the present study, the study area had a significant impact on the prevalence of obesity. Among the studies reporting genetic differences between the Amami and the mainland populations,^23^^,^^31^^,^^32^ two used data from the J-MICC study.^23^^,^^32^ Nishiyama et al found a low but significant level of genetic differentiation between the mainland population and the population of the Amami Islands,^32^ while Wakai et al reported that some polymorphisms showed a substantial difference in minor allele frequency among the participating cohorts.^23^ They proposed that genetic variation among the study areas should be considered when analyzing the data from the J-MICC study. According to the Japanese Single Nucleotide Polymorphisms (JSNP) database, the frequency of *hOGG1* genotypes of Ser/Ser, Ser/Cys, and Cys/Cys was reported for 18%, 59%, and 23% of participants, respectively.^33^ In this study, the genotype frequency of Ser/Ser, Ser/Cys, and Cys/Cys polymorphisms in mainland Japan was 22.7%, 50.7%, and 26.6%, while those on Amami Island were 13.4%, 41.2%, and 45.4%, respectively. We found that the variation in the *hOGG1* genotype Cys/Cys frequency between the Amami and the mainland could lead to a false-positive result if the study area was not considered. This is known as confounding by population stratification,^34^ which needs to be carefully considered in genetic epidemiology, even in the relatively homogeneous Japanese population. A significantly higher BMI in the Amami area may reflect population differences in genetic or environmental factors; therefore, further investigation is needed.

This study has several methodological limitations. First, the cross-sectional nature of our study limits our ability to determine causation, even though we excluded participants who were on medication for type 2 diabetes. In addition, we did not have appropriate replication data accompanying this study. Second, although measuring fat accumulation using computed tomography scans or echograms is ideal, we used BMI to evaluate obesity. Misclassification of obesity may have therefore occurred; however, misclassification of obesity would be expected to lower estimations for the association. Third, there may be intrinsic information bias in our assessments of lifestyle-related factors, such as dietary and family history. However, if any misclassification were present, it would be non-differential by the *hOGG1* genotype and would likely underestimate the true associations. Finally, although we adjusted for potential confounding factors in the multivariate analysis, residual confounding factors by known or unknown risk factors may have been present.

In conclusion, these results suggest that the *hOGG1* Ser/Cys genotype may have some influence on obesity, although its contribution is smaller than the influence of the study area. While our study found no associations of this genotype with BMI or FBG levels, we did find evidence of confounding by population stratification for these associations. This report may provide important information for genetic association analysis in the Japanese population.

## ONLINE ONLY MATERIAL

Abstract in Japanese.
