# Supplementary material for: Associations Between hOGG1 Ser326Cys Polymorphism and Increased Body Mass Index and Fasting Glucose Level in the Japanese General Population
Source: J Epidemiol. 2014 Sep 5;24(5):379–84. doi: 10.2188/jea.JE20140002 (PMC4150008; doi:10.2188/jea.JE20140002)
Supplement: Abstract in Japanese. [file je-24-379-s001.pdf]

日本人における *hOGG1* Ser326Cys 遺伝子多型と BMI、空腹時血糖との関連

原めぐみ[1]、中村和代[2]、南里妃名子[3]、西田裕一郎[1]、菱田朝陽[4]、川合沙世[4]、浜島信之[5]、喜多義邦[6]、鈴木貞夫[7]、Eva Mariane Mantjoro[8]、大中佳三[9]、上村浩一[10]、松井大輔[11]、尾瀬功[12]、三上春夫[13]、久保充明[14]、田中英夫[12]、日本多施設共同コーホート研究（ジェイミック　スタディ）グループ

[1]佐賀大学医学部社会医学講座予防医学，[2]聖マリア学院大学看護学部

[3]昭和大学医学部衛生学公衆衛生学講座公衆衛生学，[4]名古屋大学大学院医学系研究科予防医学，[5]名古屋大学大学院医学系研究科社会生命科学講座医療行政学

[6]滋賀医科大学公衆衛生学，[7]名古屋市立大学大学院医学研究科公衆衛生学

[8]鹿児島大学大学院医歯学総合研究科国際島嶼医療学, [9]九州大学大学院医学研究院予防医学, [10]徳島大学大学院ヘルスバイオサイエンス研究部予防医学

[11]京都府立医科大学大学院医学研究科地域保健医療疫学, [12]愛知県がんセンター研究所疫学･予防部,

[13]千葉県がんセンター研究局がん予防センター

[14]理化学研究所ゲノム医科学研究センター

背景：DNA 修復酵素であるヒト 8—オキソグアニン DNA グリコシラーゼ *(hOGG1)*の Ser326Cys 遺伝子多型はインスリン抵抗性や２型糖尿病との関連が示唆されているが、その機序については明らかでない。近年、動物実験において *OGG1* の遺伝子多型と肥満の有意な関連が報告されたが、人での報告はほとんどない。本研究の目的は、*hOGG1* の遺伝子多型と BMI および空腹時血糖との関連を調べることである。

方法: 日本多施設共同コーホート研究（ジェイミック　スタディ）に参加した 40～69 歳の男女 1,793

人の資料及び試料を用いて横断研究を実施した。*hOGG1* の遺伝子多型は、Multiplex PCR-based Invader assay 法により決定した。重回帰分析、共分散分析、ロジスティック回帰分析を用いて交絡因子を調整し検討を行った。

結果 : 性、年齢、摂取エネルギー、飲酒、喫煙、身体活動量、糖尿病の家族歴を補正しても、Cys アレル保有者では BMI、空腹時血糖、総コレステロールが有意に高い値を示した。BMI と Cys アレルの有意な関連は、さらに空腹時血糖と総コレステロールの値を調整しても認められたが、調査地域（奄美地域か、それ以外の地域か）で調整すると有意な関連は検出されなくなった。Cys/Cys 型では高い BMI（>27.5 kg/m^2)との有意な関連がみられたが、調査地域を調整すると関連は弱まり、有意差も検出されなくなった。

結論 : *hOGG1* の遺伝子多型と肥満の関連に対し、日本人に限った研究においても調査地域による交絡が存在することが示された。

キーワード : *hOGG1*、肥満、BMI、空腹時血糖、遺伝子多型、調査地域
